# Evaluation of analytical performance and clinical applicability of the UriSed Mini, a semi-automated urine analyzer for cell counting in cerebrospinal fluid

**DOI:** 10.11613/BM.2026.020705

**Published:** 2026-04-15

**Authors:** Oana Roxana Oprea, Ana-Maria Fotache, Ion Bogdan Mănescu, Minodora Dobreanu

**Affiliations:** 1 Department of Laboratory Medicine, Faculty of Medicine, George Emil Palade University of Medicine, Pharmacy, Science, and Technology of Targu Mures, Targu Mures, Romania; 2 Clinical Laboratory, Emergency County Clinical Hospital of Targu Mures, Targu Mures, Romania

**Keywords:** cerebrospinal fluid (CSF), UriSed Mini, cell counting, WBC counting

## Abstract

**Introduction:**

Cerebrospinal fluid (CSF) analysis is essential in diagnosing central nervous system pathologies. While manual cell counting by optical microscopy is the gold standard, it is labor-intensive and time-consuming, posing challenges particularly in urgent cases, automated hematology analyzers offer alternatives but come with limitations. The aim of the study was to evaluate the performance of the UriSed Mini instrument for CSF in accordance with ICSH guidelines.

**Materials and methods:**

A total of 77 CSF patient samples were analyzed by both methods from September 2023 to September 2024. Following the International Council for Standardization in Hematology guidelines, the UriSed Mini was evaluated for short- and long-term imprecision, limit of blank, limit of detection and analytical measurement range. Quantitative comparison between the two methods was conducted using Bland-Altman and Passing-Bablok analyses. Qualitative agreement was assessed through inter-rater agreement testing.

**Results:**

The UriSed Mini successfully met all performance criteria, both in comparison to the manufacturer’s claims and in the context of practical clinical requirements. While statistically significant differences were observed in the quantitative comparison (0.6 cells/µL), these discrepancies are unlikely to have clinical significance at the medical decision-making level. The qualitative comparison demonstrated excellent agreement, with a weighted kappa coefficient of 0.96 (95% confidence interval: 0.89-1.00).

**Conclusions:**

This study demonstrates that the UriSed Mini meets analytical standards for CSF cell counting and aligns closely with optical microscopy at clinically relevant thresholds.

## Graphical abstract

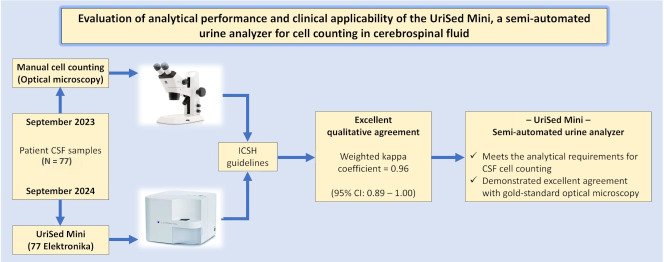

## Highlights

• This study first shows that the UriSed Mini aligns with optical microscopy for cerebrospinal fluid

• The inter-rater test showed a weighted kappa of 0.96, indicating strong agreement

## Introduction

In emergency settings, cerebrospinal fluid (CSF) collection and biochemical/cellular analysis are recommended for patients suspected of acute bacterial or viral meningitis and other types of neurological diseases, as these represent the most crucial steps in confirming or excluding the diagnosis (*1*-*3*). In the United States alone, over 300,000 lumbar punctures are performed annually in both adult and pediatric populations (*4*). This high volume of samples translates into a substantial workload for laboratories, requiring significant resources in terms of both sample handling and time. Although manual cell counting by optical microscopy is the gold standard, it is labor-intensive and time consuming, presenting challenges especially in emergency cases (*5*). Automated hematology analyzers offer an alternative but come with limitations, such the need for microscopic review, depending on their characteristics and limitations (*6*). Thus, an automated method for cell counting in CSF (and other body fluids) is highly advantageous, as it minimizes variability and, crucially, reduces the time needed to obtain results. According to the International Council for Standardization in Hematology (ICSH) guidelines, validation of a new automated cell counting method for body fluids necessitates an extensive performance evaluation and comparison with the manual gold standard (*7*).

The aim of the study was to evaluate the performance of the UriSed Mini instrument for CSF in accordance with ICSH guidelines.

## Materials and methods

The study was conducted from September 2023 to September 2024 in the clinical laboratories of a tertiary hospital, using residual CSF samples. Prior to initiation, ethical approval was obtained from the hospital’s Ethics Committee (18062/30.06.2023).

All samples underwent initial assessment by manual microscopic evaluation and were subsequently analyzed within two hours of collection using the UriSed Mini (77 Elektronika, Budapest, Hungary). Samples were collected in clear tubes (Beckton Dickinson, Franklin Lakes, USA), 2 ml from each patient. All samples were collected by the clinicians in our hospital through lumbar punction. Imprecision, both within-run and between-run, was evaluated, and additional parameters were assessed, including the limit of blank (LoB), limit of detection (LoD), and analytical measurement range (AMR) for both white blood cells (WBCs) and red blood cells (RBCs). Furthermore, a comparative analysis was performed between the UriSed Mini and the manual counting method for the CSF samples.

The UriSed Mini (77 Elektronika, Hungary) is a semi-automated instrument for analyzing urinary sediment, requiring only a small sample volume (170 µL), which can be directly pipetted into the test cuvette. According to manufacturer’s protocol, the cuvette undergoes centrifugation at 2000 rpm for 10 seconds. In RUO (*research use only*) body fluid mode, sample analysis occurs in two stages. In the first stage, which parallels urinary sediment analysis, 170 µL of the sample is processed, generating 45 microscopic images. In the second stage, the sample is manually diluted with a Body Fluid Stain, and another 170 µL of the mixture is analyzed in a second cuvette, producing an additional 45 images. These 90 images *per* sample are then analyzed to calculate final results in cells/µL. The instrument quantifies WBCs, RBCs and other nucleated cells. For samples with more than 1.5 WBC/µL after staining, the instrument also reports the percentages of polymorphonuclear cells (PMN) and mononuclear cells. Using the Research Only Software, the UriSed Mini may analyze five types of body fluids: CSF, pleural fluid (PLE), ascitic fluid (ASC), pericardial fluid (PER), continuous ambulatory peritoneal dialysis (CAPD). However, the software has not yet been validated for clinical use.

### Microscopic evaluation

For the manual microscopic evaluation of body fluids, samples were analyzed by two experienced laboratory technicians using Fuchs-Rosenthal counting chambers. In samples with a high RBC count, Turk solution was applied to lyse the RBCs, and the final result was adjusted based on the dilution factor.

### Imprecision assessment

For the short-term imprecision study, 10 replicates of the quality control (QC) material (both negative and positive controls) were measured on the same day. The QC material for body fluids is a synthetic product provided by the instrument manufacturer (Spinalscopics, Quantimetrix, Irvine, USA; Body fluid stain, 77 Elektronika, Budapest, Hungary). In addition, the short-term imprecision was also assessed on patient samples by performing 10 replicates of a CSF sample within two hours of sample collection. The patient samples were analyzed using 90 images *per* sample, following the manufacturer’s protocol.

For the long-term imprecision study, 20 replicates of the QC material were measured over the course of one month, following the body fluid protocol (90 images *per* determination). Due to the inability to store patient samples without risk of cell degradation, patient samples were not included in the long-term imprecision assessment. For leukocyte and erythrocyte counts in body fluids, no biological variation–based quality specifications are available. According to CLSI H56-A, laboratories are expected to establish their own performance goals during method validation (*6*). In line with Westgard’s recommendations, an imprecision of up to 20% is considered acceptable for analytes with inherently high variability, provided clinical decision-making is not compromised (*8*).

### Limit of blank and limit of detection

To determine the LoB, a CSF patient sample with no detectable WBCs or RBCs on manual examination was measured 11 times on the same day. The sample diluent, considered an acellular fluid, was also measured 10 times over three days.

For the LoD, four CSF patient samples with low counts of WBCs and RBCs (< 2 WBC/µL and < 3 RBC/µL), as determined by manual examination, were tested in six replicates each. The results were then compared to the LoD specified by the manufacturer to evaluate alignment with the stated performance criteria.

### Analytical measurement range

To establish the AMR, two CSF samples were selected: one with a high RBC count and one with a high WBC count, in alignment with the manufacturer’s declared AMR range of 2-1200 WBC/µL and 3-1800 RBC/µL. Acceptable precision criteria were set as follows: for points around 15 particle/µL, a standard deviation (SD) of < 5% was considered acceptable. For about 150 particle/µL, a coefficient of variation (CV) of < 15% was required. These acceptability criteria were the manufacturer’s claim stated by the manufacturer.

Each sample was diluted with saline to obtain three distinct concentration levels, with three replicate measurements performed at each concentration. The mean, SD and CV% were calculated for each point, and the data were analyzed using a correlation test to assess linearity across the concentration range. For the correlation test performed, the mean of the three replicates was used.

### Comparison with manual method

Each sample was counted manually using Fuchs-Rosenthal chambers and subsequently measured on the UriSed Mini, with both assessments completed within two hours of sample collection. A quantitative comparison was conducted to evaluate numerical consistency, alongside a qualitative comparison for clinical relevance. For the qualitative analysis, WBC results were categorized into two groups: samples with ≤ 5 WBC/µL and those with > 5 WBC/µL. Agreement between methods was assessed using the kappa coefficient.

### Statistical analysis

Descriptive statistics were applied to determine the LoB and LoD. For the analytical measurement range, data sets were compared using Pearson’s correlation test. The distribution of variables was assessed using the D’Agostino-Pearson test. To compare UriSed Mini results with manual counts, Bland-Altman analysis and Passing-Bablok regression were conducted, alongside an inter-rater agreement test (kappa) with linear weights for the qualitative assessment. All statistical analyses were performed using MedCalc Statistical Software, version 20.104 (MedCalc Software Ltd, Ostend, Belgium), with statistical significance set at P < 0.05.

## Results

The results of the imprecision assessment are shown in Table 1 (short-term imprecision) and Table 2 (long-term imprecision). For WBCs in CSF, we obtained a within-run SD of 0.35 (CV 19.45%), though this was at a mean count of 1.8 WBC/µL, below the LoQ specified by the UriSed Mini manufacturer; and a SD of 2.1 (CV 8.18%) with mean values of 25 RBC/µL. As anticipated, CV% values decreased as cell counts increased in both samples and controls. In an internal study of the manufacturer (unpublished data), the imprecision results were: SD 0.9 (CV 11%) at 8.4 WBC/µL, SD 5.0 (CV 7%) at 74.8 WBC/µL.

**Table 1:** Results of short-term imprecision for UriSed Mini with quality control material and patient sample

**Body fluid type**	**Mean**	**SD**	**CV (%)**
Negative control (RBC/µL)	12.80	1.59	12.46
Positive control (RBC/µL)	75.62	5.93	7.85
Negative control (WBC/µL)	4.29	0.77	17.86
Positive control (WBC/µL)	50.45	4.17	8.27
Patient CSF (RBC/µL)	25.62	2.10	8.18
Patient CSF (WBC/µL)	1.80	0.35	19.45
Negative control and positive control - synthetic QC sample Spinalscopics, Quantimetrix (10 replicates each). The accepted criteria for imprecision were < 20%. The target mean and range of the QC samples are the same as for the long-term imprecision study (see Table 2). RBC - red blood cell. WBC - white blood cell. SD - standard deviation. CV - coefficient of variation. CSF - cerebrospinal fluid.			

**Table 2:** Results of long-term imprecision evaluation for UriSed Mini with quality control material

**Body fluid type**	**Mean**	**SD**	**CV (%)**	**Target mean (range)**
Negative control (RBC/µL)	12.28	2.41	19.64	6.60 (0.00-13.30)
Positive control (RBC/µL)	70.73	13.70	19.32	64.40 (27.70-101.10)
Negative control (WBC/µL)	5.23	0.82	15.71	8.88 (0.00-17.70)
Positive control (WBC/µL)	47.39	4.50	9.41	58.80 (12.20-105.00)
Negative control and positive control - synthetic QC sample Spinalscopics, Quantimetrix (20 replicates each). The accepted criteria for imprecision were < 20%. RBC - red blood cell. WBC - white blood cell. SD - standard deviation. CV - coefficient of variation.				

For the LoB, across all replicates, no WBCs or RBCs were found in the CSF patient sample that was negative on manual investigation, and neither in the sample diluent. It is important to note that in the UriSed Mini, each sample is analyzed in a single-use cuvette, and sampling is performed manually by the user using a disposable pipette tip. Additionally, the dilution and staining of the sample are done separately in single-use laboratory tubes, eliminating the risk of sample diluent contamination. As a result, we did not perform a carry-over study. For the LoD, the results of the initial examination and of consequent diluted samples, are shown in Table 3 for RBCs and WBCs.

**Table 3:** Limit of detection for RBC-CSF and WBC-CSF

**RBC-CSF**				
**Sample**	**Mean (RBC/µL)**	**SD**	**95% CI**	**Manufacturer claim**
A	0.66	0.90	0.13-1.18	
B	1.7	0.98	0.73-2.66	
C	0.75	0.51	0.01-1.02	0.93
D	0.97	0.66	0.32-1.62	
**WBC-CSF**				
**Sample**	**Mean (WBC/µL)**	**SD**	**95% CI**	**Manufacturer claim**
A	0.46	0.33	0.19-0.72	
B	0.40	0.38	0.06-0.73	
C	0.90	0.60	0.41-1.38	0.68
D	0.90	0.88	0.22-1.50	
RBC - red blood cell. WBC - white blood cell. SD - standard deviation. CI - confidence interval. CSF - cerebrospinal fluid.				

**Figure 1:**
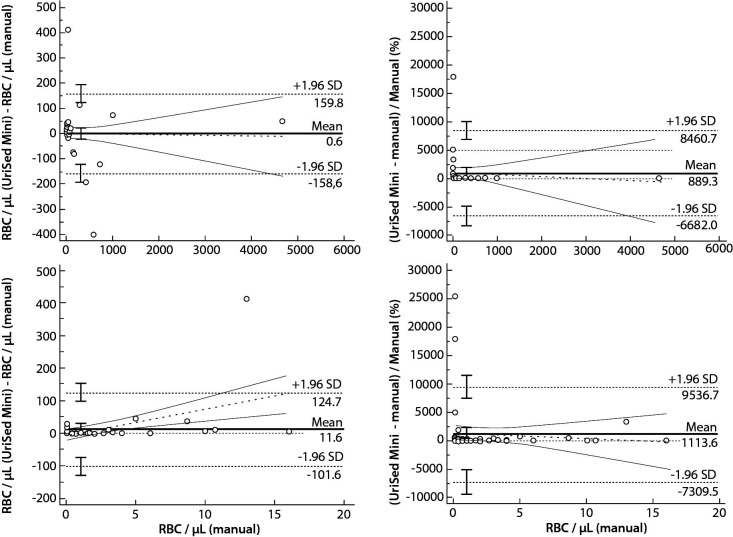
Bland-Altman plots comparing RBC counts obtained with the UriSed Mini (test method) and manual microscopic counting (reference method), shown in absolute units (RBC/µL, left) and percentages (%, right). Upper panels: full RBC range (N = 65); lower panels: 0-20 WBC/µL range (N = 52).

**Figure 2:**
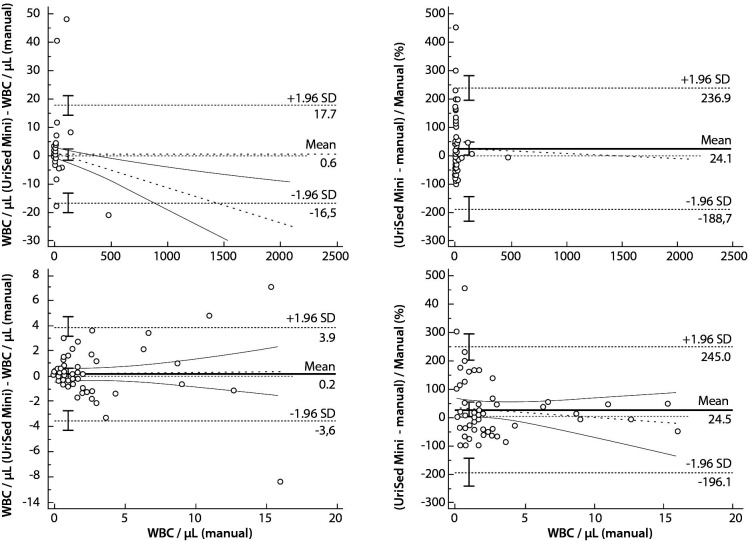
Bland-Altman plots comparing WBC counts obtained with the UriSed Mini (test method) and manual microscopic counting (reference method), shown in absolute units (WBC/µL, left) and percentages (%, right). Upper panels: full WBC range (N = 77); lower panels: 0-20 WBC/µL range (N = 68).

A wide variety of samples (N = 77) were used for the comparison between UriSed Mini and the manual investigation, with values ranging between 0-2100 WBC/µL and 0-4718/µL for RBCs. The distribution test had the following results: manual RBC count, P < 0.001; UriSed Mini RBC count, P < 0.001; manual WBC count, P < 0.001; and UriSed Mini WBC count, P < 0.001. Result of the Bland-Altman analysis is shown in Figures 1 and 2.

**Figure 3:**
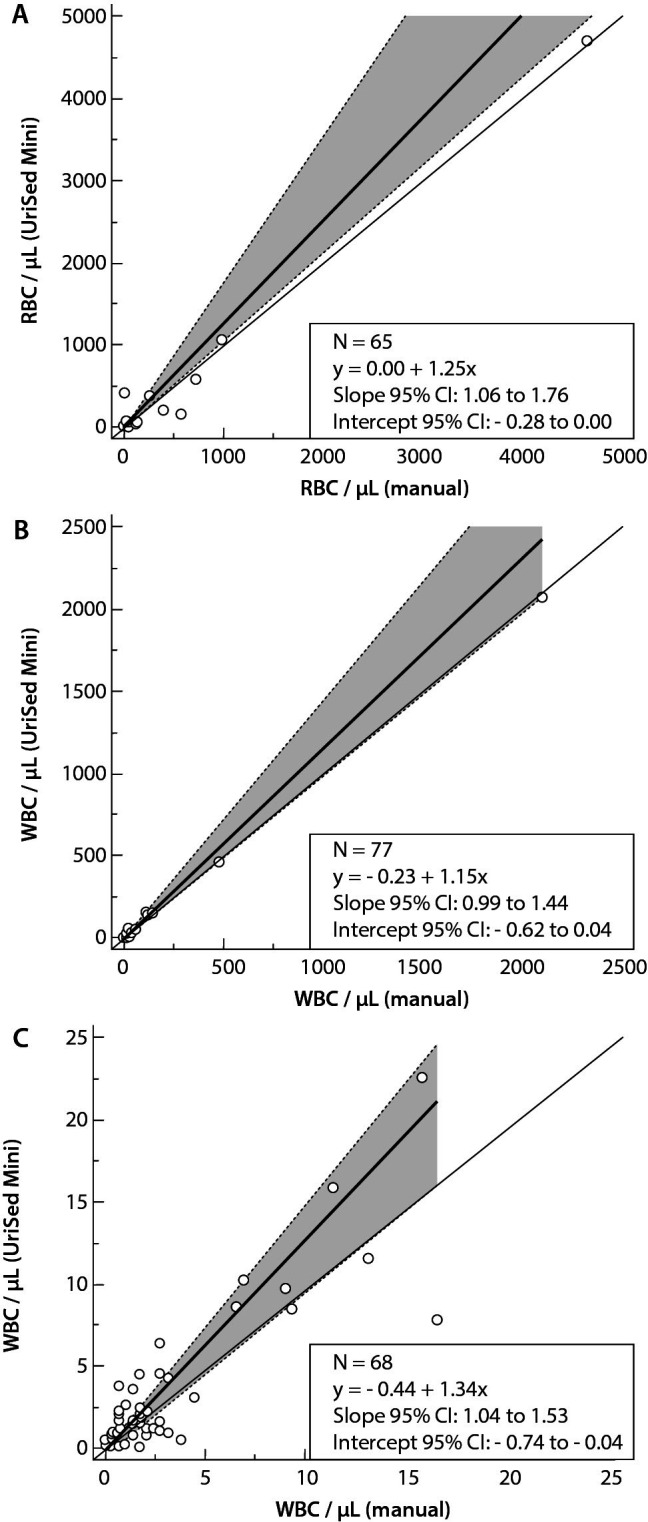
Passing-Bablok regression plots obtained by comparing cell counts obtained from UriSed Mini (test method) and manual microscopic counting (reference method). A) RBC count (whole range, N = 65). B) WBC count (whole range, N = 77). C) WBC count (range 0-20 WBC/µL, N = 68).

The Passing Bablok regression for RBC (y = 0 + 1.25 x), WBC - whole range of samples (y = - 0.23 + 1.15 x) and WBC < 20 cells/µl (y = - 0.44 + 1.34 x) results are shown in Figure 3. Before the regression analysis, linearity was tested and no significant deviation was found for either WBCs (P = 0.810) or RBCs (P = 0.310).

For the qualitative comparison, out of the 77 CSF samples, 59 were included in the negative category (≤ 5 WBC/µL) by both methods, 17 were included in the positive category (> 5 WBC/µL) by both methods, and one sample was discordant, being classified as positive by the automated count and negative by the manual count. The inter-rater agreement test showed a weighted kappa of 0.96 (95% CI: 0.89-1.00), which shows a very good agreement between methods.

## Discussion

In this study, an evaluation of the UriSed Mini urine analyzer for CSF samples was performed. The instrument’s imprecision (CV < 20%) was assessed using both patient samples and QC material. For LoB, LoD and AMR patient samples were used. Agreement between UriSed Mini and the manual method (gold standard) was evaluated with 77 patient samples.

The within-run imprecision observed for RBC and WBC counts in both QC materials and body fluid samples remained below the predefined 20% limit, consistent with acceptable performance ranges reported for automated body fluid counting on hematology analyzers. Previous evaluations of Sysmex XN-series instruments and other automated platforms have documented similarly low imprecision values for total nucleated cell and RBC counts, supporting the analytical stability seen in our study (*9*-*11*). As commonly reported, higher CVs occurred at low cell concentrations; however, the corresponding absolute variation was too small to affect clinical interpretation, particularly around the established CSF decision thresholds where the presence of any RBCs or a WBC count above 5/µL is clinically meaningful (*1*).

Between-run imprecision was likewise < 20% across QC levels, consistent with findings for the UriSed3 in urinary sediment analysis and aligned with the study’s predefined criteria (*12*).

The limits of blank observed in the literature for automated analyzers vary widely but generally fall within low ranges for both RBCs and WBCs (*5*). The manufacturer’s LoD for the UriSed Mini also fit within the 95% confidence intervals obtained in our CSF sample measurements and were comparable to LoD values reported for Sysmex instruments, while some analyzers such as Iris did not report LoD for body fluids (*5*). These concordances support the reliability of the UriSed Mini at the low cell concentrations most relevant for CSF analysis.

Analytical measurement range assessment showed performance compatible with the manufacturer’s specifications, and correlation coefficients were similar to those previously reported for automated body fluid analyzers (Table 4) (*5*). Agreement analyses using Bland-Altman and Passing-Bablok regression indicated small, systematic differences between methods. For RBCs, the UriSed Mini showed a slight proportional overestimation compared with manual counting, most evident at low concentrations, although the magnitude of the bias remained below clinically relevant levels. For WBCs, a systematic underestimation at higher counts and a slight overestimation at low counts were observed, but again without compromising clinical interpretation, particularly in the critical 0-5 WBC/µL range.

**Table 4:** Analytical measurement range for RBC-CSF and WBC-CSF

**RBC-CSF**			
**Sample**	**Mean (RBC/µL)**	**SD (RBC/µL)**	**CV (%)**
Undiluted	1568.87	46.78	2.98
Dilution 1	779.63	25.36	3.25
Dilution 2	503.23	2.79	0.56
Dilution 3	379.40	5.54	1.46
Manufacturer claim	Analytical measurement range: 3-1800 RBC/µL		
Results	*r* = 1.00 (95% CI 0.99-1.00), P < 0.001		
**WBC-CSF**			
**Sample**	**Mean (WBC/µL)**	**SD (WBC/µL)**	**CV (%)**
Undiluted	1206.00	27.79	2.26
Dilution 1	805.40	10.51	1.31
Dilution 2	240.67	7.20	2.99
Dilution 3	28.07	5.18	18.47
Manufacturer claim	Analytical measurement range: 2-1200 WBC/µL		
Results	*r* = 1.00 (95% CI 0.99-1.00), P < 0.001		
RBC - red blood cell. WBC - white blood cell. SD - standard deviation. CV - coefficient of variation. CSF - cerebrospinal fluid.			

Given that CSF interpretation relies primarily on categorical thresholds-especially the cut-off of > 5 WBC/µL to indicate potential CNS pathology-the high categorical agreement observed between manual counts and the UriSed Mini is clinically reassuring (*1*, *2*). Only one discordant sample was identified among 77 tested, and the weighted kappa coefficient (0.96) demonstrated near-perfect agreement, comparable to performance levels reported for other automated body fluid analyzers.

Automated hematology instruments provide reliable overall performance but may show limited accuracy at low cell counts, a limitation noted in previous evaluations. In contrast, the UriSed Mini demonstrated strong performance in this range, likely aided by its low detection limits and integrated optical verification function. The ability to visually confirm cell morphology and detect bacteria directly on the instrument provides an additional advantage compared with analyzers lacking optical microscopy capabilities.

In summary, the UriSed Mini showed analytical performance comparable to that reported for established automated body fluid analyzers, with reliable agreement in the clinically critical low-count range (*5*, *9*-*12*). These findings support its suitability as an alternative to manual microscopy for routine CSF cell counting.

While numerous studies have explored the performance of automated hematology analyzers for body fluid cell counting, this study uniquely utilized a urine analyzer for this purpose. This novel approach, coupled with the adherence to ICSH guidelines for performance evaluation, represents a key strength of the study. Last but not least, the use of disposable cuvettes in the case of UriSed equipment, compared to the extensive washing in BF mode of the hematology instrument is less time consuming. The primary limitation was the relatively small sample size. However, these findings serve as a preliminary investigation, providing a foundation for a subsequent study with a larger cohort of CSF samples to further validate the method. Because of the low numbers of WBC in our samples we were not able to evaluate the performances of the instrument for differentiated leukocyte count (neutrophils, mononuclear *etc.*). To evaluate this, a high number of WBCs in BF are needed.

To fully evaluate the performances of the analyzer, a multicentric study should be performed in which multiple body fluid types and samples from a wide range of pathologies may be included.

Automated cell counters offer a compelling alternative or complement to traditional manual methods, bringing increased speed and consistency to cell counting in body fluids. This study is the first to demonstrate that the UriSed Mini, an automated urine analyzer, not only meets all analytical performance criteria for body fluid cell counting but also aligns closely with optical microscopy, the gold standard, regarding clinically relevant thresholds. Further validation studies are recommended for broader clinical application, and laboratories are advised to conduct their own performance evaluations. Nevertheless, the UriSed Mini, which functions as a “hybrid” between automated counting and optical microscopy, appears to be a viable method for CSF cell counting in both routine and especially emergency testing.

## Data Availability

The data generated and analyzed in the presented study are available from the corresponding author on request.
